# Supplementary material for: A unified formulation of dichroic signals using the Borrmann effect and twisted photon beams
Source: Sci Rep. 2018 May 21;8:7941. doi: 10.1038/s41598-018-23627-5 (PMC5962556; doi:10.1038/s41598-018-23627-5)
Supplement: Supplementary file 1 — Supplementary Information [file 41598_2018_23627_MOESM1_ESM.pdf]

Supplementary information

A unified formulation of dichroic signals using the Borrmann effect and twisted photon beams

Stephen P Collins[1] and Stephen W Lovesey[1, 2]

1. Diamond Light Source Ltd, Oxfordshire OX11 0DE, UK

2. ISIS Facility, STFC, Oxfordshire OX11 0QX, UK

A scattering length f derived from the Kramers-Heisenberg dispersion function is,

$$f = -\, \mathrm{F}/(\mathrm{E} - \Delta + i\Gamma/2), \qquad \text{(A1)}$$

where E is the photon energy, and Δ and Γ are the energy and total width of the atomic resonance labelled η. The amplitude F = $\{V'_{2\eta}\ V_{1\eta}\}$ where matrix elements of the electron-photon interaction operator V between states 1 & 2 and the intermediate state $V'_{2\eta}$ & $V_{1\eta}$ account for photon creation and annihilation [32]. V is proportional to the photon polarization vector $\boldsymbol{\epsilon}$. In consequence, F contains a product $\epsilon'_\alpha \epsilon_\beta$ that is usefully expressed through a tensor product,

$$X^{K''}_{Q''} = \sum_{\alpha,\beta} \epsilon'_\alpha \epsilon_\beta\, (1\alpha\, 1\beta \,|\, K''Q'') = \{\boldsymbol{\epsilon}' \otimes \boldsymbol{\epsilon}\}^{K''}_{Q''}. \qquad \text{(A2)}$$

The Clebsch-Gordan coefficient and Wigner 3-j symbol in (A2) are standard with,

$$(a\alpha b\beta \,|\, KQ) = (-1)^{-a+b-Q} \sqrt{(2K+1)} \begin{pmatrix} a & b & K \\ \alpha & \beta & -Q \end{pmatrix}.$$

One finds $X^{K''}_{-Q''} = (-1)^{K''+Q''} (X^{K''}_{Q''})^*$ and,

$$X^0_0 = -\,(1/\sqrt{3})\, [\boldsymbol{\epsilon}' \bullet \boldsymbol{\epsilon}],\ \mathbf{X}^1 = (i/\sqrt{2})\, [\boldsymbol{\epsilon}' \times \boldsymbol{\epsilon}],\ X^2_0 = (1/\sqrt{6})\, [3\epsilon'_0\epsilon_0 - \boldsymbol{\epsilon}' \bullet \boldsymbol{\epsilon}],$$

$$X^2_{+1} = (1/\sqrt{2})\, [\epsilon'_0\epsilon_{+1} + \epsilon'_{+1}\epsilon_0],\ X^2_{+2} = \epsilon'_{+1}\epsilon_{+1}. \qquad \text{(A3)}$$

Our Cartesian coordinate scheme is depicted in Fig. 1a. For a normal dichroic signal and linear polarization the required values of $\mathbf{X}^{K''}$ use $\boldsymbol{\epsilon}' \bullet \boldsymbol{\epsilon} = 1$, with $X^2_0 = -\,(1/\sqrt{6})$, $X^2_{+1} = 0$ and $X^2_{\pm 2} = 1/2$. Upon averaging over circular polarization in the primary photon beam $(\mathbf{X}^1)_{av} = -\,(1/\sqrt{2})\, \hat{\mathbf{q}}\, P_2$, where P_2 is a Stokes parameter for circular polarization [4] and $\hat{\mathbf{q}} = (0, 0, 1)$.

We calculate the the value of F produced by the interaction of twisted radiation with ions, and adopt the standard assumptions. A dipole matrix element of the type needed in F has been calculated by Alexandrescu *et al.* with the same assumptions [11]. Radiation is treated classically in the paraxial approximation. The spatial spread of electronic states is assumed to be small compared to the waist w of the twisted beam. In these circumstances the electric field $\mathbf{E}$ can be expressed in terms of solid spherical-harmonics $\mathfrak{R}^l_n(\mathbf{b})$ with an argument $\mathbf{b}$ proportional to the transverse component $\mathbf{r}_\perp$ of the position of an electron. The angular

orientation of **b** is carried by a spherical harmonic in $\mathfrak{R}^l_n(\mathbf{b})$. For a transverse component $\mathbf{r}_\perp$ the topological charge and its projection must satisfy $l + n$ even and,

$$\mathbf{E} \propto \boldsymbol{\epsilon}\, \mathfrak{R}^l_n(\mathbf{b}), \qquad \text{(A4)}$$

with $n = \pm l$ and $\mathbf{b} = \mathbf{r}_\perp/w$. The polarization vector $\boldsymbol{\epsilon}$ and $\mathbf{r}_\perp$ are confined to the plane normal to the direction of propagation of the beam, which is taken to be the z-axis in Fig. 1 of the main text. The proportionality factor in (A4) is purely real. The corresponding dipole interaction operators are,

$$\mathrm{V} \propto \mathbf{r} \bullet \boldsymbol{\epsilon}\, \mathfrak{R}^l_n(\mathbf{b}), \text{ and } \mathrm{V}' \propto \mathrm{V}^* \text{ using a polarization vector } \boldsymbol{\epsilon}', \qquad \text{(A5)}$$

with the electron position $\mathbf{r} \propto \boldsymbol{\mathfrak{R}}^1(\mathbf{r})$ measured relative to an origin at **R**, giving $w\mathbf{b} = \mathbf{R}_\perp + \mathbf{r}_\perp$. For a topological charge $l = 1$ the interaction V is evidently a sum of $(r_\alpha\, \mathbf{R}_\perp)$ and $(r_\alpha\, \mathbf{r}_\perp)$. Application of the triangle-rule for the product of two dipoles, $(r_\alpha\, \mathbf{r}_\perp)$ say, tells us that the it can be represented by the sum of a scalar, dipole and a quadrupole $\mathfrak{R}^2_\mu(\mathbf{r})$. An expansion of $\mathfrak{R}^l_n(\mathbf{b})$ in products $\mathfrak{R}^a_\alpha(\mathbf{R}_\perp/w)$ & $\mathfrak{R}^c_\chi(\mathbf{r}_\perp/w)$ with $c \le l$, where $a + \alpha$ & $c + \chi$ are even integers, facilitates the evaluation of matrix elements for $l \geq 2$.

Returning to the amplitude, we consider a typical term in F that is diagonal with respect to the topological charge. The product of the interesting matrix elements is,

$$\mathrm{F} = \langle \lambda | \mathbf{r} \bullet \boldsymbol{\epsilon}' \{\mathfrak{R}^l_n(\mathbf{b})\}^* | \eta \rangle \langle \eta | \mathbf{r} \bullet \boldsymbol{\epsilon}\, \mathfrak{R}^l_n(\mathbf{b}) | \lambda' \rangle = \sum_{k',k} \sum_{K',K''} \sum_{K,Q} (2k' + 1)\,(2k + 1)\, \Upsilon^K_Q(k', k)$$

$$\times\, (-1)^{n+Q} \begin{pmatrix} 1 & l & k' \\ 0 & 0 & 0 \end{pmatrix} \begin{pmatrix} 1 & l & k \\ 0 & 0 & 0 \end{pmatrix} \{\Pi^{K'} \otimes \mathrm{X}^{K''}\}^K_Q\, [(2K' + 1)(2K'' + 1)]^{1/2} \begin{Bmatrix} K' & K'' & K \\ l & 1 & k' \\ l & 1 & k \end{Bmatrix}, \qquad \text{(A6)}$$

where $\Pi^{K'}_{Q'} = (ln\ l{-}n \,|\, K'\ Q')$ that is different from zero when $Q' = 0$. We assume that the intermediate state is spatially isotropic, to a good approximation, leaving it characterized solely by total angular momentum J_c that resides in the atomic tensor $\Upsilon^K_Q(k', k)$. This simplification of the product of matrix elements is not necessary, however. A general result, with all quantum labels of the intermediate state, is given by Balcar and Lovesey together with steps in its reduction to (A6) [22]. The spherical tensor $\Upsilon^K_Q(k', k)$ is also a function of quantum labels in $|\lambda\rangle$ and $|\lambda'\rangle$ that belong to the ground-sate of an ion, whereas intermediate states $|\eta\rangle$ are virtual and do not obeys Hund's rules. Not shown explicitly in (A6) is a product of reduced matrix elements (RMEs) for spherical harmonics $[(l_v \| C(k') \| l_c)(l_c \| C(k) \| l_v)]$, where l_v and l_c are angular momenta for the valence and core states, respectively. An RME of this type is different from zero for $l_v + l_c + k$ even, say, so the aforementioned product is different from zero for $(k + k')$ even. The 3-j symbols in (A6) are different from zero for $(l + k')$ and $(l + k)$ odd integers, which leads to the same condition on $(k + k')$. Variables in each row and each column of the 9-j symbol are subject to a triangular condition.

The Clebsch-Gordan coefficient $\Pi^{K'}_0 = (ln\ l{-}n \,|\, K'\ 0) = (-1)^{K'} (l{-}n\ ln \,|\, K'\ 0)$, i.e., $\Pi^{K'}_0$ is an odd function of n for K' odd and an even function of n for K' even. In an experiment this

finding translates to a powerful selection rule on atomic information available from a difference ΔF of dichroic signals produced with opposite handedness in the photon beam. The selection rule becomes even more influential when it is combined with specific polarization in the primary beam, e.g., $K'' = 1$ for circular polarization.

The photon tensor for a twisted beam ($l = 1$) and circular polarization can be different from zero for zero projection ($Q = 0$), and we write it as $H^K_0(n, P_2)$. One finds,

$$H^K_0(+,+) = -\sqrt{(2K+1)} \begin{pmatrix} 2 & 2 & K \\ -2 & 2 & 0 \end{pmatrix} = (-1)^K \, H^K_0(-,-),$$

and,

$$H^K_0(+,-) = H^K_0(-,+) = -(1/6)\sqrt{(2K+1)} \begin{pmatrix} 2 & 2 & K \\ 0 & 0 & 0 \end{pmatrix},$$

is different from zero for K even. Specific values of $H^K_0(n, P_2)$ appear in Table 4. The result $H^4_0(+,+) = H^4_0(+,-)$ accounts for the absence of a hexadecapole in the difference signal listed in Table 1.

For dichroism created with topological charge $l = 1$, application of the triangular condition shows that the rank $K' = 0, 1, 2$. Discussions in the main text concern quadrupole events and $k = k' = 2$ in (A6). Electronic multipoles then obey $0 \leq K \leq 4$, and in the application to dichroic signals $\Upsilon^K_Q(k', k)$ reduces to a multipole $\langle T^K_Q \rangle$ associated with the electronic ground sate, even though it depends on the total angular momentum of the core state J_c. The spherical tensor operator is Hermitian and $\langle T^K_Q \rangle^* = (-1)^Q \langle T^K_{-Q} \rangle$. The atomic multipole is completely specified by its RME (equation (73) in reference [4]), multiplied by $(l_v \| C(2) \| l_c)^2$ in an application [4]. The RME uses a standard unit-tensor that contains fractional parentage coefficients, and the unit-tensors have been listed for d and f atomic states [20]. A dependence on J_c creates sum rules for integrated signals [2, 4].

32. Berestetskii, V. B., Lifshitz, E. M. & Pitaevskii, L. P. *Course of Theoretical Physics* vol. 4 2nd ed. (Pergamon Press, 1982)

Process	Tensor	Prefactor	Projection (Q)				
			0	± 1	± 2	± 3	± 4
Normal absorption	X_Q^0	$-\frac{1}{\sqrt{3}}$	1				
E1-E1	X_Q^1	0					
Linear polarization (x)	X_Q^2	$-\frac{1}{\sqrt{6}}$	1	0	$-\sqrt{3/2}$		
Normal absorption	H_Q^0	$\frac{1}{2\sqrt{5}}$	1				
E2-E2	H_Q^1	0					
Linear polarization (x)	H_Q^2	$-\frac{1}{2\sqrt{14}}$	1	0	$\sqrt{3/2}$		
	H_Q^3	0					
	H_Q^4	$-\frac{2}{\sqrt{70}}$	1	0	$-\frac{\sqrt{10}}{4}$	0	0
Normal absorption	X_Q^0	$-\frac{1}{\sqrt{3}}$	1				
E1-E1	X_Q^1	$-\frac{1}{\sqrt{2}}$	± 1	0			
Circular polarization P_2 ±1	X_Q^2	$-\frac{1}{\sqrt{6}}$	1	0	0		
Normal absorption	H_Q^0	$\frac{1}{2\sqrt{5}}$	1				
E2-E2	H_Q^1	$-\frac{1}{2\sqrt{10}}$	± 1	0			
Circular polarization $P_2 = \pm 1$	H_Q^2	$-\frac{1}{2\sqrt{14}}$	1	0	0		
	H_Q^3	$\frac{1}{\sqrt{10}}$	± 1	0	0	0	
	H_Q^4	$-\frac{2}{\sqrt{70}}$	1	0	0	0	0

Table 2. Photon tensor components $\mathbf{X}^K$(dipole transitions, equation (A2)) and $\mathbf{H}^K$(quadrupole transitions, equations (1) or (4)) for normal absorption with linear polarization along the x-axis using $\boldsymbol{\epsilon} = \boldsymbol{\epsilon}' = (1, 0, 0)$. Polarization vectors $\boldsymbol{\epsilon} = (1, i, 0)/\sqrt{2}$ & $\boldsymbol{\epsilon}' = (1, -i, 0)/\sqrt{2}$ for right-handed circular polarization with Stokes parameter $P_2 = +1$. The photon wavevector is along the z-axis in Fig. 1a ($\hat{\boldsymbol{q}} = (0,0,1)$).

Process	Tensor	Prefactor	Projection (Q)				
			0	± 1	± 2	± 3	± 4
Borrmann Effect (E1-E1)	X_Q^0	0					
Borrmann Effect	H_Q^0	$\frac{-1}{2\sqrt{5}}$	1				
(E2-E2)	H_Q^1	0					
Linear polarization	H_Q^2	$\frac{1}{\sqrt{14}}$	1	0	0		
	H_Q^3	0					
	H_Q^4	$\frac{-1}{2\sqrt{70}}$	1	0	0	0	$-\frac{\sqrt{70}}{2}$

Table 3. The photon tensor $H^K{}_Q$ is derived from either (1) or (4). Photon tensor components for the Borrmann case, with linear polarization along the x-axis using $\boldsymbol{\epsilon} = \boldsymbol{\epsilon}' = (1, 0, 0)$, $\hat{\mathbf{q}} = \boldsymbol{\kappa}_b = (0, 1, 0)$ and $\hat{\mathbf{q}}' = -\boldsymbol{\kappa}_b$.

Process	Tensor	Prefactor	Projection (Q)				
			0	$\pm$	±2	±3	±4
OAM (E1-E1, |n|>0)	X_Q^K	0					
OAM (E2-E2, $n=\pm1$)	H_Q^0	$\frac{-7}{12\sqrt{5}}$	1	0			
E2-E2	H_Q^1	$\frac{1}{\sqrt{10}}$	±1	0			
Linear polarization	H_Q^2	$\frac{-5}{6\sqrt{14}}$	1	0	$-\frac{\sqrt{6}}{5}$		
	H_Q^3	$\frac{1}{2\sqrt{10}}$	±1	0	$\mp\sqrt{5/6}$	0	
	H_Q^4	$\frac{-1}{\sqrt{70}}$	1	0	$-\frac{\sqrt{10}}{4}$	0	0
OAM circular polarization	H_Q^0	$\frac{-1}{\sqrt{5}}$	1				
E2-E2	H_Q^1	$\frac{2}{\sqrt{10}}$	±1	0			
$P_2=\pm1$ $n=\pm1$	H_Q^2	$\frac{-2}{\sqrt{14}}$	1	0	0		
	H_Q^3	$\frac{1}{\sqrt{10}}$	±1	0	0	0	
	H_Q^4	$\frac{-1}{\sqrt{70}}$	1	0	0	0	0
OAM circular polarization	H_Q^0	$\frac{-1}{6\sqrt{5}}$	1				
E2-E2	H_Q^1	0					
$P_2=\pm1$ $n=\mp1$	H_Q^2	$\frac{1}{3\sqrt{14}}$	1	0	0		
	H_Q^3	0					
	H_Q^4	$\frac{-1}{\sqrt{70}}$	1	0	0	0	0

Table 4. The photon tensor H^K_Q for the OAM (twisted beam) case, with linear polarization (top), and circular polarization parallel and antiparallel to the OAM (middle and bottom). The effective wave vectors for winding number $n = (\pm1)$, are $\hat{\mathbf{q}} = \boldsymbol{\kappa}_t$ and $\hat{\mathbf{q}}' = -(\boldsymbol{\kappa}_t)^*$ with $\boldsymbol{\kappa}_t = (-i, 1, 0)/\sqrt{2}$ for $n = +1$, and $\boldsymbol{\kappa}_t = (i, 1, 0)/\sqrt{2}$ for $n = -1$. Circular polarization $P_2 = \pm1$ with vectors $\boldsymbol{\epsilon} = (1, i, 0)/\sqrt{2}$ & $\boldsymbol{\epsilon}' = (1, -i, 0)/\sqrt{2}$ for right-handed circular polarization $P_2 = +1$.
